# X-linked agammaglobulinemia diagnosed late in life: case report and review of the literature

**DOI:** 10.1186/1476-7961-6-5

**Published:** 2008-06-02

**Authors:** Justin R Sigmon, Ehab Kasasbeh, Guha Krishnaswamy

**Affiliations:** 1 Division of Allergy and Immunology, Department of Internal Medicine, East Tennessee State University, College of Medicine, Johnson City, Tennessee 37614, USA

## Abstract

**Background:**

Common variable immune deficiency (CVID), one of the most common primary immunodeficiency diseases presents in adults, whereas X-linked agammaglobulinemia (XLA), an inherited humoral immunodeficiency, is usually diagnosed early in life after maternal Igs have waned. However, there have been several reports in the world literature in which individuals have either had a delay in onset of symptoms or have been misdiagnosed with CVID and then later found to have mutations in Bruton's tyrosine kinase (BTK) yielding a reclassification as adult-onset variants of XLA. The typical finding of absent B cells should suggest XLA rather than CVID and may be a sensitive test to detect this condition, leading to the more specific test (Btk mutational analysis). Further confirmation may be by mutational analyses.

**Methods:**

The records of 2 patients were reviewed and appropriate clinical data collected. BTK mutational analysis was carried out to investigate the suspicion of adult-presentation of XLA. A review of the world literature on delayed diagnosis of XLA and mild or "leaky" phenotype was performed.

**Results:**

2 patients previously diagnosed with CVID associated with virtual absence of CD19^+ ^B cells were reclassified as having a delayed diagnosis and adult-presentation of XLA. **Patient 1**, a 64 yr old male with recurrent sinobronchial infections had a low level of serum IgG of 360 mg/dl (normal 736–1900), IgA <27 mg/dl (normal 90–474), and IgM <25 mg/dl (normal 50–415). **Patient 2**, a 46 yr old male with recurrent sinopulmonary infections had low IgG of 260 mg/dl, low IgA <16 mg/dl, and normal IgM. Mutational analysis of BTK was carried out in both patients and confirmed the diagnosis of XLA

**Conclusion:**

These two cases represent an unusual adult-presentation of XLA, a humoral immunodeficiency usually diagnosed in childhood and the need to further investigate a suspicion of XLA in adult males with CVID particularly those associated with low to absent CD19^+ ^B cells. A diagnosis of XLA can have significant implications including family counseling, detecting female carriers, and early intervention and treatment of affected male descendents.

## Background

Primary humoral immune deficiency disorders are characterized by defects in antibody production leading to significantly weakened humoral immunity. These patients are highly susceptible to recurrent bacterial infections, bacteremia, and sepsis resulting in high mortality rates. Among these disorders there is heterogeneity of clinical manifestations and immunological defects observed. Differentiating one humoral immune deficiency syndrome from another requires thorough immunological evaluation in a timely fashion since early diagnosis and treatment are essential to patient outcomes and survival. In recent years, advances in genetic mutational analysis have allowed physicians to more accurately diagnose patients that present with recurrent infections and are suspected of having an underlying primary immune deficiency. X-linked agammaglobulinemia (XLA), although a disorder of infants and children, sometimes may be diagnosed late in life. In this instance, it may be easily confused for another disorder-common variable immune deficiency (CVID). Though the clinical and prognostic outcomes may be considered to be similar in the two disorders, the genetic basis is different, leading one to evaluate family transmission more aggressively or consider gene therapy as an option in one or more of these conditions.

We present two cases of XLA diagnosed late in life, and review the clinical features and outcomes of similar cases described in the world literature. CVID is one of the most common primary immunodeficiency diseases requiring medical treatment with a reported prevalence of 1 in 50,000 in the general population and usually presents in adulthood[1]. XLA on the other hand is a recessive primary immunodeficiency disorder with a reported prevalence of 1/10,000 in the general population[1]. XLA is associated with mutations in the Bruton's tyrosine kinase (Btk) gene, which is integral to B cell signaling and maturation. In patients with XLA, typically immunological evaluation shows marked deficiency or absence of CD19^+ ^B lymphocytes and severely decreased levels of all isotypes of immunoglobulins, however wide variability in clinical presentation among families with XLA have been observed. In contrast to CVID, XLA is caused by a congenital defect of B cell development and most often presents during infancy after maternal Igs have dissipated, however, there have been several cases previously described in which individuals had late onset of chronic infection or were misdiagnosed with CVID and later found to have Btk mutations [2,3]. Some of these cases highlight the heterogeneity of this disorder and may be related partially to the extent of the genetic defect, associated B cell dysfunction and apoptosis, or other poorly defined modifying factors. Some of these patients with XLA presenting late in life may represent a "leaky" or mild phenotype [4] as reviewed later in the Discussion. In the two cases of XLA diagnosed as an adult and described by us in this report, mutational analysis demonstrated hemizygous Btk mutations. This led us to subsequently reclassify the patients as having an adult-presentation of XLA rather than CVID.

## Methods

Approval was made by the institutional review board and the records of two patients were reviewed and appropriate immunological data collected. Peripheral blood lymphocyte and immunoglobulin enumerations were determined by flow cytometric analysis. Informed consent was obtained from both patients for Btk mutational analyses which were carried out by Correlagen Diagnostics laboratory in Worcester, Massachusetts. A review of the world literature for all cases of adult presentation of XLA was performed using a PubMed search with MeSH terms/keywords: XLA, X-Linked Agammaglobulinemia, atypical XLA, adult and "leaky" or "mild" XLA.

### Case Report

#### Patient 1

A 53-year-old male presented to the allergy and immunology clinic for evaluation of hypogammaglobulinemia, recurrent upper respiratory infections, recurrent bronchitis, pneumonias, and urinary tract infections. Review of his past medical history showed that at age 19 he was diagnosed with bronchiectasis requiring a left lower lobe lung resection and at age 23, he was diagnosed at Georgia Tech as having hypogammaglobulinemia. Two years prior to this diagnosis, his teenage brother was diagnosed as having agammaglobulinemia and still receives intravenous immunoglobulin. He received immunoglobulin preparations intermittently after diagnosis of hypogammaglobulinemia which seemed to decrease the number of infections. His last pneumonia was at age 20 and he has had recurrent episodes of sinobronchial infections several times per year since. His personal medical history also revealed that he had pansinusitis and surgery for double sinus windows around the age of 32. He also had a positive history for gastroesophageal reflux disease, prostatitis, and hyperlipidemia. Physical exam yielded no relevant findings other than the scar of lobectomy and expiratory wheezing. No splenic enlargement or lymphadenopathy was seen.

Laboratory results at his initial presentation revealed a normal white blood cell count, low levels of serum IgG, IgA, and IgM. Flow cytometric analysis showed zero CD19^+ ^B cells, normal CD4^+ ^T cells, and elevated CD8^+ ^T cells (Table 1). Tests for anti-IgA antibody were compatible with lack of anti-IgA antibodies. Serum protein electrophoresis revealed no evidence of paraproteinemia. The initial diagnosis was determined to be CVID and intravenous immunoglobulin (IGIV) was started at a dose of 400 mg/kg of Gamimune every 4 weeks as a prophylactic measure. This was deemed appropriate since he was hypogammaglobulinemic, demonstrated significantly impaired functional responses to pneumococcal serotypes, had a history of severe sinopulmonary disease including pansinusitis and bronchiectasis requiring surgical resection, and to prevent further progression to severe chronic obstructive lung disease. He continued to get regular IVIG infusions and maintained trough levels of IgG above the lower end of normal (550 mg/dL).

**Table 1:** Results of immunological evaluation*

	**Patient 1**	**Patient 2**
Age	64	46
		
WBC (cells/uL)	8.3 (3.2–9.8)	6.9 (5.0–10.2)
Granulocytes (%)	59 (42–75)	68 (45–75)
Lymphocytes (%)	28 (20–51)	18 (20–50)
Monocytes (%)	9.5 (0–12)	12 (0–8)
		
IgM (mg/dL)	25	95 (50–300)
IgG (mg/dL)	360	260 (565–1765)
IgA (mg/dL)	27	16 (40–350)
		
C3 (mg/dL)	ND	129 (85–200)
C4 (mg/dL)	ND	23 (14–53)
CH50 (U/ml)	ND	51 (22–60)
		
CD4+ (cells/uL)	1023 (720–1440)	624 (575–1070)
CD8+ (cells/uL)	979 (315–788)	477 (190–860)
NK (cells/uL)	ND	135 (150–880)
CD19+ (cells/uL)	0 (113–495)	0 (70–300)
		
SPEP	Hypogammaglobulinemia	Hypogammaglobulinemia
Pneumococcal responses	Impaired	Impaired

Abbreviations: SPEP, serum protein electrophoresis; WBC, white blood cells; NK, natural killer;

* Values in parentheses represent reference ranges

At age 64 he was reevaluated and found to have significantly decreased CD19^+ ^B cells. This finding along with his history of recurrent infection and a family history of dysgammaglobulinemia prompted the decision to investigate the possibility of a Btk mutation. Informed consent was obtained and Btk mutational analyses were conducted. The results demonstrated a hemizygous point mutation associated with a single amino acid change in the pleckstrin homology (PH) domain of the Btk gene (Table 2) consistent with a diagnosis of XLA.

**Table 2:** Results of Btk mutational analysis

	**Patient 1**	**Patient 2**
Nucleotide change	c.83G>A	c.1223T>C
Amino acid change	p.Arg28His	p.Leu408Pro
Domain	PH	SH1
Zygosity	Hemizygous	Hemizygous

PH, Pleckstrin Homology; SH1, Src homology 1

#### Patient 2

A 42-year-old male presented to the allergy and immunology clinic for evaluation of recurrent infections and suspected immunodeficiency. From his personal medical history it was revealed that he had onset of infections at age 3 with a spinal meningitis followed by recurrent episodes of bronchitis, pneumonitis, and hospitialization four times for pneumonia. Physical exam yielded no relevant findings.

Laboratory results at his initial presentation revealed low serum levels of IgG and IgA, with normal levels of IgM. His flow cytometric analysis showed zero C19^+ ^B cells, normal CD4^+ ^and CD8^+ ^T cell counts, and low NK (natural killer cell) cell counts (Table 1). His total white blood cell count was normal, as were polymorphonuclear cells, but his lymphocytes were slightly decreased, and monocytes were above reference range (Table 1). Anti-nuclear antibody and rheumatoid factor were both negative. Serum protein electrophoresis showed hypogammaglobulinemia and no evidence of paraproteinemia. The total serum protein and albumin levels were normal (Table 1). His CH-50, C3, and C4 levels were within reference range, but his pneumococcal responses were significantly impaired.

A suspicion of X-linked agammaglobulinemia given his unusual absence of CD19^+ ^B cells prompted further evaluation for Btk mutations. At this time he was treated for his current sinobronchial infection and classified as having common variable immunodeficiency. IVIG was initiated at a dose of 400 mg/kg of Gamimune every 4 weeks as a prophylactic measure. During this time he maintained trough levels of IgG at or above 500 mg/dL and reported having fewer sinobronchial infections requiring antibiotics. Four years later, at the age of 46, he was reevaluated and informed consent was obtained and Btk genetic mutational tests were conducted. The results demonstrated a hemizygous point mutation associated with a single amino acid change in the Src homology 1 (SH1) domain of the Btk gene confirming a diagnosis of XLA (Table 2). His nephew was recently diagnosed with hypogammaglobulinemia and is being evaluated for XLA.

## Discussion

Infantile and congenital immune deficiencies can present in middle age or in the elderly and can be mistaken for a variety of conditions such as atopy and CVID. Further testing and evaluation may be required in such situations as identification of a genetic and molecular defect will make family screening easier, will allow potential gene therapy in the future, and will also educate the patient about their condition.

We present 2 cases of XLA diagnosed late in life in which both patients were initially diagnosed as having CVID. These patients presented with a history of recurrent upper and lower respiratory tract infections requiring antibiotics since childhood, but managed to survive into adulthood without any acute life-threatening infections despite having no replacement of immunoglobulins. Flow cytometric analysis demonstrated absent CD19^+ ^B cells and normal CD4^+ ^T cell numbers in both patients, but normal to elevated numbers of CD8^+ ^T cells. These findings along with poor specific antibody responses to pneumococcal antigens lead to genetic mutational analysis for Btk mutations. Both patients were found to have hemizygous Btk mutations, in the absence of mutations described with CVID (such as TACI gene mutations). Since treatment of patients with XLA or CVID is primarily to replace immunoglobulin and antibiotic therapy as needed, both patients continued to receive IGIV at optimal doses and have had moderate clinical improvement and reduction of infections. We review below other reported incidences of XLA presenting at an advanced age. These cases were detected using a PubMed search of the world literature as described under Methods.

### Review of adult diagnosed XLA in the world literature

A review of the world literature revealed 16 cases of adult presentation of XLA prior to these 2 cases which we present (Table 3) [2-12]. Most of these patients were diagnosed during evaluation for recurrent pneumonia, sinusitis, and otitis media infection and subsequently diagnosed by Btk mutational analysis with XLA in adulthood. 5 patients were noted to have been previously diagnosed with CVID and later reclassified as atypical variants of XLA. These patients ranged from 21 to 60 years of age and had IgG levels ranging between 20 and 773 mg/dL prior to initiation of treatment with IGIV. The low to normal levels of IgG in many of these patients may partially explain why symptoms in many of these patients were mild or subclinical until diagnosis in adulthood. Marked variation in the effects of each Btk mutation on the production of functional Btk protein may account for the presence of small numbers of immunoglobulin producing CD19^+ ^B cells and a marginal ability to evade severe infection through childhood in these patients.

**Table 3:** Clinical data and Btk mutations of 16 patients reported in the world literature with atypical XLA.

		Serum Ig level (mg/dL)*			Btk Mutation		
			
**Patient no.**	**Age (yrs)**^ϕ^	**IgG**	**IgA**	**IgM**	**Nucleotide Change**	**Domain**	**Reference**
P1	51	401	<7	15	567C>A	TH	12
P2	26	169	8	7	UD	UD	
P3	25	773	UD	1	UD	UD	4
P4	34	420	UD	UD	UD	UD	
P5	27	635	<5	11	Glu605stop	SH1	9
P6	40	<20	<20	22	G>A^ψ^	NA	5
P7	39	220	UD	UD	W563L	SH1	
P8	60	429	7	14	994C>T	SH2	6
P9	27	132	7	17	230C>T	PH	10
P10	21	35	8	29	1630A>G	SH1	8
P11	32	462	<8	<7	227T>C	PH	
P12	32	702	185	<25	1706G>A	SH1	
P13	28	454	95	38	UD	UD	11
P14	27	346	16	8	1705C>T	SH1	
P15	24	NA	0	1	1942-1943del AG	SH1	
P16	31	527	8	30	UD	UD	

UD, Undetermined; NA, Not applicable

* Serum immunoglobulins reported at the age of diagnosis

ϕ Age at diagnosis

ψ Splice site mutation at the 3' end of intron 13: IVS 13 -1 G>A

Kornfeld et al described a case of extreme variation in a three-generation family in which the proband, a 51 year old male with recurrent sinusitis, was found to have atypical XLA as was a nephew whom was diagnosed upon contracting pseudomonas bacteremia at age 10 months and then died at age 8 from encephalitis [13]. This case demonstrates the clinical variability of a XLA within a family in which typical and atypical variants exist and the need for maternal screening and early treatment of affected offspring.

### Molecular Defect

XLA is caused by an arrest in B cell development associated with mutations in the Btk gene which has been mapped to Xq21.3-q22. BTK is a member of the Tec family of nonreceptor tyrosine kinases and is expressed throughout B-cell development from CD34^+ ^CD19^+ ^pro-B cells to mature B cells. Other cells express Btk including erythroid precursors, mast cells, monocytes, myeloid cells, megakaryocytes, and platelets, however its expression is absent in T and natural killer cells [1]. Btk functions to transduce signals from the B cell immunoglobulin receptor (BCR) and absence of Btk has been shown to halt normal B cell development at the pre-B transitional cell stage with premature induction of apoptosis. Btk signaling is integral to the progression of pre-B1 cells to pre-B2 cells. Btk promotes phosphorylation of residues in phospholipase C gamma (PLCg) which in turn activates 1,4,5-triphosphate (IP3) and diacylglycerol (DAG). These second messengers ultimately promote movement of intracellular calcium and activation of protein kinase C (PKC). PKCβ activation is key to the activation of NF-κB and subsequent signaling needed for cell survival. Without sufficient Btk protein, these pro-survival signals are not made and the pre-B cells undergo immature apoptosis. In patients with XLA, studies have shown pro-B and pre-B1 cells to comprise more than 80% of the bone marrow B cell population compared with less than 20% in normal individuals [1].

The Btk genome sequence consist of 19 exons and mutations have been reported in all five of the domains of the Btk gene including the Pleckstrin Homology (PH), Tec homology (TH), Src homology 1 (SH1), Src homology 2 (SH2), and Src homology 3 (SH3). The PH domain is considered to be the most distinct and has crystal structure similar to many other signaling proteins. The PH domain consists of a positively charged ligand-binding pocket that binds phosphatidylinositol lipids. This binding is necessary to promote Btk translocation and localization to the cell membrane [1]. Mutations in this domain such as the hemizygous point mutation in our patient may inhibit the signaling needed for recruitment of Btk to the cell membrane where it functions to transduce signals from the B cell receptor (BCR) of pre-B cells. The TH domain contains of a proline rich region which interacts with SH3 domains and may have a regulatory function on other Tec family members [14]. The SH domains of Btk are very similar to classical Src tyrosine kinase domains. SH1 functions as the catalytic kinase domain and the SH2 and SH3 domains are responsible for binding and interacting with tyrosine-phosphorylated proteins and polyproline motifs [1]. Mutations in each of these domains have been described in patients with XLA, but no correlation between onset and severity of specific mutations in these patients have been described.

### Mutations of Btk

According to Valiaho et al and their 2006 review of the online mutation database for Btk mutations, BTKbase, 1,111 patient entries have been compiled from 973 unrelated families with 602 unique molecular events. Of all these mutations 40% were missense mutations leading to amino acid substitutions, premature stop codons, and exon skipping, while the remainder consisted of 17% nonsense, 20% deletions, 7% insertions, and 16% splice-site mutations [15]. The distribution of mutations is relatively proportional to the size of each of the five domains. Our two cases had mutations in the PH and SH1 domains (Table 2). From review of the world literature, 16 cases of XLA with diagnosis delayed to adulthood were found. Mutations were found in four of the five domains including 2 PH domain mutations, 1 SH2 domain mutation, 1 TH domain mutation, 6 SH1 domain mutations, and 6 did not specify a domain. 8 of the 10 cases which specified a domain were point mutations and 2 were deletions (Table 3). There appears to be no direct correlation between mutations in any specific domain with adult presentation of XLA. Interestingly Saffran et al described an SH2 domain mutation which allowed normal levels of Btk transcript to be produced and encoded an unstable protein in an individual with atypical XLA suggesting that subtle mutations may block one pathway in the Btk signaling cascade while other alternate pathways may continue to function [6]. Variation in severity of specific mutations may account for "mild" phenotypic variants of XLA allowing certain individual's CD19^+ ^B cells to survive long enough to produce sufficient amounts of immunoglobulins to avoid life-threatening infections in childhood. Some of the cases diagnosed as adults may represent "mild" phenotypes of XLA despite having significantly low to absent B cells. Noordzij et al suggest that these milder clinical phenotypes may be associated with BTK splice-site mutations which produce lower levels of wild-type BTK transcripts [4].

### Differentiating XLA and CVID

Knowledge of the key characteristics of XLA and CVID may assist in the differentiation of these two clinically similar diseases (Table 4). XLA and CVID are both humoral immunodeficiencies that can manifest similar clinical presentations when encountering a patient in their second to sixth decade as seen in our two cases. Distinguishing between XLA and CVID in a patient can have significant implications when considering the morbidity of affected males and their descendents. With the benefit of technological advances in recent years, screening female carriers and other male relatives, and genetic counseling now serve pivotal roles in the healthcare of a family with XLA. Table 4 discusses these differences in detail.

**Table 4:** Key characteristics of XLA and CVID

	**XLA**	**CVID**
**Age of onset**	usually by 9–18 months	usually 2nd – 4th decade
		
**Family Hx of immunodeficiency**	usually +ve	variable*
		
**Inheritance**	x-linked recessive	variable
		
**Diagnosis**		
Lymph nodes/tonsils	absent tonsillar tissue	normal tonsillar tissue
CD19^+ ^B cell numbers	markedly decreased/absent	normal/low
CD4^+ ^T cell numbers	Normal	Variable**
CD8^+ ^T cell numbers	Normal	Variable**
CD4^+ ^CD8^+ ^ratio	Variable	often decreased
Specific Antibody titers	absent	decreased/absent
Mutations reported	Btk	TACI, ICOS, BAFF-R, CD19^+^
		
**Common Complications**	Infections	Infections
	Allergy/Atopy	Allergy/Atopy
	CEMA, VAPP	-----
	Autoimmunity	Autoimmunity
	Malignancy	Malignancy
		
**Treatment**	**IGIV**	**IGIV**
	Symptomatic care	Symptomatic care***

*Some familial clustering has been described in the literature, possibly associated with Class II MHC gene complex

**CD4^+ ^and CD8^+ ^numbers may be low or normal

*** Symptomatic care includes antimicrobials, surgical drainage, nebulizer treatment for wheezing, allergy management, avoidance, nutrition, etc.

Abbreviations: TACI, Transmembrane activator and calcium-modulator and cyclophilin ligand interactor, Btk, Bruton's tyrosine kinase, ICOS, inducible costimulatory receptor, CEMA, chronic enteroviral meningoencephalitis, VAPP, vaccine-associated paralytic poliomyelitis, IGIV, intravenous immunoglobulin.

## Conclusion

In conclusion, we present two males diagnosed in adulthood with CVID whom upon further investigation by BTK mutational analysis were found to have XLA. These two cases demonstrate delayed diagnosis and adult presentation of XLA which is usually diagnosed in the first decade of life in male children with recurrent bacterial infections. DNA sequencing of the BTK gene from each patient showed mutations in the PH domain of patient 1 and the SH1 domain of patient 2. Each of these mutations had been previously reported in the BTKbase online registry. Prior to these two patients, 16 other patients were reported in the world literature with adult presentation of XLA. Any male patient who presents with recurrent infections, hypogammaglobulinemia, and low to absent CD19^+ ^B cells should be suspected of having XLA. In atypical cases such as these two that we present, the virtual absence of CD19^+ ^B cells may be a sensitive test to differentiate XLA from CVID, which may lead to the more specific genetic mutational analysis for Btk mutations.

## Competing interests

The authors declare that they have no competing interests.

## Authors' contributions

JS carried out the literature review, drafted the manuscript, and configuration of tables and figures, EK assisted in data collection, design of tables, and literature review, GK supplied the manuscript outline and reviewed the manuscript for final submission.
